# Pericardial windows have limited diagnostic success

**DOI:** 10.1186/s13019-018-0774-x

**Published:** 2018-07-18

**Authors:** Lindsay Volk, Hirohisa Ikegami, Leonard Y. Lee, Anthony Lemaire

**Affiliations:** Division of Cardiothoracic Surgery, Department of Surgery, RUTGERS-Robert Wood Johnson Medical School, 125 Paterson Street, New Brunswick, NJ 08903 USA

**Keywords:** Effusion, Tamponade, Drainage

## Abstract

**Background:**

Pericardial effusion (PE) is a common finding in patients who have chronic cardiac failure, who had undergone cardiac surgery, or who have certain other benign and malignant diseases. Pericardial drainage procedures are often requested for both diagnostic and therapeutic purposes. The perceived benefit is that it allows for diagnosis of malignancy or infection for patients with PEs of unclear etiology. The purpose of the study is to determine the diagnostic yield of surgical drainage procedures.

**Methods:**

We conducted a retrospective chart review of patients who underwent surgical drainage procedures of PEs from July 1st, 2011 to January 1st, 2017 at a single institution. The variables included data on preoperative, intraoperative, and postoperative findings; morbidity; and survival.

**Results:**

A total of 145 patients with an average age of 61 ± 5 and primarily men (53%) were evaluated. All of the surgical drainage procedures were performed through the sub-xiphoid approach. Twenty-five of the 145 patients (17.2%) had diagnostic findings in either the pericardial tissue or fluid. The cytology alone was diagnostic in 4.8% (*N* = 7) of patients with mixed findings including adenocarcinoma of the lung and breast. The pathology was diagnostic for cancer in 1.4% (*N* = 2) of patients with Melanoma and Lung cancer identified. The cytology and pathology were concordant in 4.0% (*N* = 6) identifying cancers that included mesothelioma and adenocarcinoma. Infection was identified in the pericardial fluid in 6.9% (*N* = 10) of the patients.

**Conclusion:**

Surgical pericardial drainage procedures allow for removal of PE that may lead to tamponade physiology and potential mortality. Although there is therapeutic benefit from these procedures there is only a small diagnostic benefit.

## Background

A pericardial effusion (PE) is a common finding in patients who have chronic cardiac failure, postoperative cardiac surgery patients, or patients who have certain infectious, benign or malignant diseases. They are often diagnosed either as incidental findings or when its related to systemic or cardiac disease [1]. The clinical spectrum of patients with pericardial effusions ranges from mild asymptomatic effusions to cardiac tamponade [2, 3]. Management is guided by the hemodynamic impact, size, presence of inflammation (i.e. pericarditis), and the etiology. Drainage of the PE is required for cardiac tamponade, symptomatic moderate to large pericardial effusions and when a bacterial or neoplastic etiology is suspected [1].

Surgical pericardial drainage procedures, “Pericardial Windows”, are often requested for both diagnostic and therapeutic purposes. The perceived diagnostic benefit is that it allows for determination of malignancy or infection for patients with PEs of unclear etiology through testing of the pericardial fluid and tissue. The purpose of the study is to determine the diagnostic yield of surgical drainage procedures.

## Methods

We conducted a retrospective chart review of patients who underwent surgical drainage procedures of PEs from July 1st, 2011 to January 1st, 2017 at a single academic medical institution. The demographic data for each patient was identified. The variables included data on preoperative, intraoperative, postoperative findings, morbidity, and survival. The indications for the drainage procedures were also identified. In each operative case a sample of at least 50 cc of pericardial fluid was sent to both cytology and microbiology labs for analysis. A large sample of pericardium was also sent to the pathology lab for testing. This study was approved by the institutional review board at Rutgers Robert Wood Johnson Medical School.

### Operative techniques

The technique for surgical drainage of PEs is primarily through the subxiphoid or thoracotomy approach. When the PE is drained through the subxiphoid approach a 3 cm incision is made at the level of the xiphoid. Electrocautery is then used to divide the subcutaneous tissue down to the xiphoid process. Next, a combination of sharp and blunt dissection is used to isolate and resect the xiphoid. The remaining sternum is elevated with a retractor and the pericardium is identified. The pericardial space is entered and the effusion is drained. The opening of the pericardium is enlarged to ensure patency and drainage. At the completion of the procedure a drain is placed within the pericardium and secured. Similarly, the pericardium can be approached by going through either the left or right pleural space. Once the pericardium is identified it is entered and the effusion drained. The method of entering the pleural space can be through an open thoracotomy or with a video assisted thorascopic (VATs) approach.

## Results

A total of 145 patients with an average age of 61 ± 5 and primarily men (53%) were evaluated. All of the surgical drainage procedures were performed through the sub-xiphoid approach. Twenty-five of the 145 patients (17.2%) had diagnostic findings in either the pericardial tissue or fluid. The cytology alone was diagnostic in 4.8% (*N* = 7) of patients with mixed findings including adenocarcinoma of the lung and breast (Table 1). The pathology was diagnostic for cancer in 1.4% (*N* = 2) of patients with Melanoma and Lung cancer identified (Table 2). The cytology and pathology were concordant in 4.0% (*N* = 6) identifying cancers that included mesothelioma and adenocarcinoma (Table 3). Infection was identified in the pericardial fluid in 6.9% (*N* = 10) of the patients (Table 4). The microorganisms were varied and included staphylococcus epidermidis and Sternotrophomas. There was 1 complication with a right ventricular perforation requiring conversion to full sternotomy and repair of the injury. There were no mortalities from the procedures.

**Table 1:** Cytology cancer type source

Adenocarcinoma (3)	Lung
Neuroendocrine (Small Cell) (2)	Lung
Adenocarcinoma	Unspecified
Adenocarcinoma	Breast
Adenocarcinoma (3)	Lung
Neuroendocrine (Small Cell) (2)	Lung
Adenocarcinoma	Unspecified
Adenocarcinoma	Breast

**Table 2:** Pathology cancer type source

Melanoma	Skin
Adenocarcinoma	Lung

**Table 3:** Cytology and pathology cancer type source

Adenocarcinoma (3)	Breast
Adenocarcinoma	Lung
Adenocarcinoma	Endometrial
Mesothelioma	Lung

**Table 4:** Microbiology organism number

*Staphylococcus epidermidis*	2
*Staphylococcus aureus*	2
Sternotrophomas Maltophilia	2^a^
Streptococcus Pneumoniae	2
Enterococcus Faecalis	1
Propionibacterium acnes	1
*Enterobacter cloacae*	1^a^

^a^One sample isolated both enterbacter cloacae and strenotrophomas maltophilia

## Discussion

The data from our study shows that surgical drainage of PE, “pericardial windows”, have low diagnostic yield with only 17.2% of the patients having informative results. Our findings also show that pericardial biopsy adds even less diagnostic yield with only 2 of the patients having positive results not otherwise yielded by cytology, while 13 patients had cytology that was diagnostic. Microbiology analysis showed to be valuable in 10 of the samples in terms of identifying infection with varied organisms. Surgical drainage procedures were a 100% successful in terms of the therapeutic benefit, but the ability to diagnosis the etiology for the effusion was less effective. These findings are not well known as often the request to perform surgical drainage of the PE is to determine the cause of the PE by either tissue or fluid. Furthermore, an often-cited reason for surgical drainage of PEs is that not only fluid can be removed but also a portion of pericardium can be analyzed as well. Our results show that this reasoning is poorly supported as less than 2% of pericardium samples produced any findings that could not also be gleaned by cytology. Although the diagnostic success is low for these procedures, fortunately the complication rate is low. The mortality of the pericardial windows in this study was 0% with only 1 patient requiring conversion from a subxiphoid approach to a full sternotomy. These findings are similar to previous studies that report low mortality and morbidity with surgical pericardial procedures [4].

The normal pericardial sac contains up to 50 mL of fluid with anything greater being a pathologic effusion. The curvilinear pressure-volume relationship of the pericardial sac determines the hemodynamic consequences of a PE and is responsible for rapidly accumulating fluid that causes cardiac tamponade. There are numerous diseases and complications which cause PEs with the most common being idiopathic pericarditis, cancer, and hemorrhage [5]. PE may be classified based on its onset (acute, subacute, or chronic when it has been present for greater than 3 months), distribution (circumferential or loculated), hemodynamic impact (none, cardiac tamponade, effusive-constrictive), and composition (exudates, transudate). PE is also distinguished by size as mild, moderate, and large based on echocardiographic assessment [1, 6].

The pathophysiology of the development of PE has been described as resulting from any pathological process causing an inflammatory process with the possible increased production of pericardial fluid. Another mechanism for the formation of pericardial fluid may be decreased reabsorption due to increased systemic venous pressure as a result of congestive heart failure or pulmonary hypertension (transudate) [7]. The most common cause is often unknown as most of the PEs are idiopathic. Although the subxiphoid approach for surgical drainage has been our preferred method there are alternative approaches. The creation of a “pericardial window” can be done through a thoracotomy [8, 9] or video-assisted approach [10, 11]. The procedure is generally low risk with a low complication rate and rare mortality.

## Conclusion

Surgical pericardial drainage procedures allow for removal of PE that may lead to tamponade physiology and potential mortality. Although there is therapeutic benefit from these procedures there is only a small (17.2%) diagnostic benefit. It is important that all clinicians be aware of these findings to appropriately set expectations.

## Abbreviation

PE: Pericardial effusion
